# Influence of cardiovascular condition on retinal and retinal nerve fiber layer measurements

**DOI:** 10.1371/journal.pone.0189929

**Published:** 2017-12-22

**Authors:** Elena Garcia-Martin, Erika Ruiz-de Gopegui, Montserrat León-Latre, Sofia Otin, Irene Altemir, Vicente Polo, Jose M. Larrosa, Marta Cipres, Jose A. Casasnovas, Luis E. Pablo

**Affiliations:** 1 Ophthalmology Department, Miguel Servet University Hospital, Zaragoza, Spain; 2 Aragon Health Research Institute (IACS-IIS Aragon), Zaragoza, Spain; 3 Health Center La Jota, Aragon Health Service, Zaragoza, Spain; 4 Department of Medicine, University of Zaragoza, Zaragoza, Spain; Bascom Palmer Eye Institute, UNITED STATES

## Abstract

**Objective:**

To assess changes in the retinal nerve fiber layer (RNFL) and macula in subjects with cardiovascular risk factors or subclinical ischemia.

**Design:**

Prospective and observational study.

**Methods:**

A total of 152 healthy men underwent cardiovascular examination, including quantification of subclinical atheroma plaques by artery ultrasound scans, blood analysis, and a complete ophthalmic evaluation, including spectral-domain optical coherence tomography. The variables registered in cardiovascular examination were quantification of classic major risk factors, subclinical atheroma plaques by artery ultrasound scans, and analytical records. The ophthalmic evaluation registered RNFL and macular thickness.

**Results:**

Mean subject age was 51.27±3.71 years. The 40 subjects without classic cardiovascular risk factors did not show differences in RNFL and macular thicknesses compared with the 112 subjects with at least one risk factor (except in sector 9 that showed higher thicknesses in subjects with ≥1 risk factor). Comparison between the group of subjects with and without atheroma plaques revealed no differences in RNFL and macular thicknesses. The sub-analysis of subjects with subclinical atheroma plaques in the common carotid artery revealed a significant reduction in central macular thickness in the left eye compared with the right eye (p = 0.016), RNFL in the superior quadrant (p = 0.007), and the 11 o’clock sector (p = 0.020). Comparison between smokers and nonsmokers revealed that smokers had significant thinning of the central macular thickness (p = 0.034), the nasal RNFL quadrant (p = 0.006), and the 3 and 5 o’clock sectors (p = 0.016 and 0.009).

**Conclusions:**

Classic cardiovascular risk factors do not cause RNFL or macular thickness reduction, but tobacco smoking habit reduces nasal RNFL thickness. Subclinical atherosclerosis in the common carotid artery associates a reduction in central macular and nasal RNFL quadrant thicknesses in the left eye compared with the right eye.

## Introduction

Numerous studies have analyzed the ability of optical coherence tomography (OCT) to detect retinal nerve fiber layer (RNFL) thickness abnormalities and changes in the macula of patients with diverse neurodegenerative diseases, as a view of the "window to the brain" [1,2]. These objective morphologic changes in the retinal and RNFL structures can be used for the diagnosis, prognosis, and follow-up of axonal neurodegeneration, and each neurodegenerative disease seems to show distinctive brain and RNFL atrophy patterns [3–5].

Chronic microvascular ischemic changes in the brain often lead to cognitive impairment and other neurologic deficits. Signs of ischemia are detected on brains scans, most typically magnetic resonance imaging. Imaging reveals small areas in the brain where blood vessels have ruptured or clotted off, leading to extremely small areas of stroke and neurodegeneration. Cognitive deficits and neurodegeneration may be associated with impaired synaptic transduction. A very high percentage of older adults may suffer from chronic microvascular changes, and RNFL defects may be the earliest signs of neurodegeneration due to ischemic events, even before the patient becomes symptomatic or brain damage can be detected by classic neuroimaging [1–3].

In the present study, we evaluated healthy subjects without a previous history of ocular or cardiovascular disease selected from the “Aragon Workers´ Health Study (AWHS)”, a longitudinal cohort study designed to identify risk factors for the development of preclinical and clinical atherosclerosis [6]. We evaluated the cardiovascular risk factors of the sample, the subclinical atheroma plaques by ultrasound scans, and specific analytical records to analyze whether those subjects with a higher cardiovascular risk or subclinical atheroma plaques had a significant reduction in macula and RNFL measurements determined by OCT.

## Methods

A total of 152 healthy male subjects were recruited and evaluated for this cross-sectional study from September 2013 to September 2015. Male staff from a manufacturing company (General Motors, Zaragoza, Spain) was offered to voluntarily take part in the study. 184 subjects were approached, 21 were not included due to not meeting the inclusion criteria.173 subjects were included in the beginning of the study, but eight subjects had to be excluded after being diagnosed with several ophthalmological conditions (chronic open angle subclinical glaucoma was detected in 5 subjects, cataract in 2 patients and a macular hole in one patient). Another three subjects dropped-out from the study. All procedures adhered to the tenets of the Declaration of Helsinki, and The Aragon regional government’s Ethics Committee for Clinical Research (CEICA) reviewed and approved this study. The informed written consent was signed by all participants before the inclusion in the study.

Cardiovascular examination for all the subjects enrolled in the study included quantification of the classic major risk factors, determination of the presence of subclinical atherosclerosis, and analytical records. The classic major and independent risk factors for cardiovascular disease are: cigarette smoking, diabetes mellitus, elevated blood pressure, and dyslipidemia. For this study, we considered elevated blood pressure as systolic blood pressure ≥140 mmHg and diastolic blood pressure ≥90 mmHg, or self-reported use of antihypertensive medication. Dyslipidemia was defined as total cholesterol ≥240 mg, low-density lipoprotein (LDL) ≥160, high-density lipoprotein (HDL) <40 mg/dl, or lipid-lowering medication. Diabetes was considered in patients with fasting plasma glucose ≥126 or taking hypoglycemic medication.

Cardiovascular risk was estimated following the guidelines of the Pooled Cohort Risk Assessment Equations, developed by the Risk Assessment Work Group, an arm of the American College of Cardiology/American Heart Association Cardiovascular Risk Guidelines. The Pooled Cohort Equations estimate the 10-year primary risk of ASCVD [atherosclerotic cardiovascular disease: nonfatal myocardial infarction (heart attack), coronary heart disease death, or stroke] among people without pre-existing cardiovascular disease between 40 and 79 years of age. People are considered to be at "elevated" risk if the Pooled Cohort Equations predicted risk is ≥ 7.5%, low risk <5%, and moderate risk 5–7.5% [7].

Subclinical atherosclerosis was defined by the presence of plaques in both carotid and both femoral arteries determined using the Philips IU22 ultrasound system (Philips Healthcare, Bothell, WA). Plaque was defined as a focal structure protruding **≥**0.5 mm into the lumen or reaching a thickness ≥50% of the surrounding intima. Examination of the carotid territory included the terminal portion (10 mm) of the common carotid, the bulb, and the initial portion (10 mm) of the internal and external carotid arteries. This method was previously published [8].

The presence of coronary calcium was evaluated following the Agatston method, and was considered as positive for any calcium score ≥1 coronary artery calcification score (CACS) [9]. The Agatston method was first described in 1990 as a novel way to measure coronary artery calcium [10]. Agatston et al. used ultra-fast computed tomography to measure total calcium scores based on the number, areas, and peak Hounsfield computed tomographic numbers of the calcified lesions detected [10].

A comprehensive ophthalmic evaluation was performed in all subjects, including best-corrected visual acuity and fundus examination under pupil dilation, to rule out the presence of any concomitant ocular pathology. Spectral domain optical coherence tomography (SD-OCT) is a noninvasive, noncontact, and highly sensitive ophthalmic diagnostic imaging technology that provides objective and precise *in vivo* measurements of macular and retinal nerve fiber layer (RNFL) thicknesses. Structural analysis of the retina was performed using SD-OCT with the Cirrus Photo OCT (Carl Zeiss Meditec Inc, Dublin, CA). OCT examination for this study included the macular (for macular thickness analysis) and RNFL protocols. The same experienced operator performed all scans and did not apply manual correction to the OCT output. We used an internal fixation target because it provides the highest reproducibility and rejected poor-quality scans prior to data analysis [11]. We assessed the image quality based on the signal strength measurement that combines the signal-to-noise ratio with the uniformity of the signal within a scan (scale 1–10, where 1 is categorized as poor image quality and 10 as excellent). We included images with a score higher than 7 for evaluation. The Cirrus OCT macular cube 512 x 128 protocol provides a macular volume measure and retinal thickness values for nine areas that correspond to the Early Treatment Diabetic Retinopathy Study chart. These areas include a central 1-mm circle representing the fovea, and inner and outer rings measuring 3 mm and 6 mm in diameter, respectively. The inner and outer rings are divided into four quadrants each: superior, nasal, inferior, and temporal. The Cirrus OCT optic disc protocol generates 200 x 200 cube images with 200 linear scans enabling analysis of the RNFL of a 6-mm^3^ volume around the optic nerve. The thickness of the retinal nerve fiber layer is measured 3.4 mm from the centre of the optic disc. This distance was chosen by Zeiss because it offers the best compromise between the thickness of the RNFL and interindividual variability.

For each scan series of RNFL measurements, we assessed the average thickness of each quadrant (superior, nasal, inferior, and temporal).

All data analyses were performed using SPSS software version 20.0 (SPSS Inc., Chicago, IL). The Kolmogorov-Smirnov test was used to assess sample distribution. For quantitative data following a parametric distribution, differences between evaluation groups were compared using Student´s t-test. For qualitative data, a chi square test was used for comparison. Correlations between groups of study were determined using Pearson’s correlation coefficient. Finally, a multivariate regression analysis was performed to identify basal RNFL or macular parameters that were predictors of presence of classic cardiovascular risk factors or subclinical atherosclerosis. A p value of less than 0.05 was considered to be statistically significant.

## Results

We enrolled 303 eyes from 152 healthy men in the study with a mean age of 51.27±3.71 years (range: 42–58 years).

In the first analysis, we divided the sample into two groups to evaluate the presence of any of the classic major cardiovascular risk factors (i.e., cigarette smoking, elevated blood pressure, diabetes mellitus, and elevated serum cholesterol level): 40 subjects (26.3%) had no classic major cardiovascular risk factors and 112 subjects (72.7%) had at least one risk factor. Mean age of the group without risk factors was 50.78±3.47 years and that of the group with risk factors was 51.47±3.78 years. Mean intraocular pressure (IOP) was 14.21±2.43 mmHg for the group without risk factors and 14.12±2.54 mmHg for the group with risk factors. The two groups did not differ significantly with regard to age (p = 0.154), or IOP values (p = 0.691). Mean values and comparison of the cardiovascular risk description (i.e., weight, height, body mass index, systolic blood pressure, diastolic blood pressure, frequency, serum glucose levels, total cholesterol levels, HDL cholesterol, LDL cholesterol, triglycerides, hematocrit, hemoglobin, leucocytes, and platelets) and OCT measurements for the groups with and without classic risk factors are shown in Table 1. OCT revealed no significant differences between the two groups with regard to the RNFL measurements or the macular structure analysis.

**Table 1:** Mean, standard deviation (SD), and comparison of cardiovascular parameters and of retinal nerve fiber layer (RNFL) and macular thickness values obtained with the Cirrus High Definition optical coherence tomography (OCT) device measured in subjects without classic major cardiovascular risk factors and subjects with at least one classic major cardiovascular risk factor. Significant differences are marked in bold. Abbreviations: LDL (low-density lipoprotein), HDL (high-density lipoprotein).

		SUBJECTS WITHOUT CARDIOVASCULAR RISK FACTORS (n = 40)		SUBJECTS WITH CARDIOVASCULAR RISK FACTORS (n = 112)		SIGNIFICANCE (P)
		Mean	SD	Mean	SD	
**Cardiovascular parameters**	Weight	80.40	10.09	84.14	11.10	**0.008**
	Height	171.53	6.78	171.79	6.49	0.759
	Body Mass Index	27.27	2.57	28.51	3.34	**0.003**
	Systolic Blood Pressure	121.53	11.41	125.65	13.96	**0.018**
	Diastolic Blood Pressure	81.40	7.36	83.57	9.08	0.054
	Frequency	67.27	11.09	69.99	12.25	0.81
	Glucose level	95.31	8.64	100.79	19.46	**0.015**
	Cholesterol total	209.52	21.87	225.72	38.40	**<0.001**
	Cholesterol HDL	55.72	14.33	51.22	10.47	**0.003**
	Cholesterol LDL	131.48	20.23	140.59	34.74	**0.027**
	Triglycerides	111.46	48.80	169.72	106.66	**<0.001**
	Hematocrit (%)	44.62	2.90	46.10	2.92	**<0.001**
	Hemoglobin	15.23	1.03	15.54	0.93	**0.015**
	Leucocytes	6.34	1.43	7.38	1.88	**<0.001**
	Platelets	243.52	38.30	242.09	61.27	0.845
**RNFL thickness**	Average	93.23	9.65	94.62	10.31	0.293
	Superior	114.53	15.43	118.78	19.32	0.076
	Nasal	74.75	11.49	72.23	14.09	0.140
	Inferior	121.89	17.91	123.19	14.39	0.518
	Temporal	62.19	9.53	64.45	10.68	0.094
	Sector 1	118.73	23.06	123.12	29.29	0.225
	Sector 2	104.10	17.92	109.10	27.39	0.128
	Sector 3	91.26	16.90	88.43	22.14	0.297
	Sector 4	60.64	11.13	59.31	11.76	0.377
	Sector 5	72.32	14.39	68.87	15.28	0.079
	Sector 6	103.62	23.44	103.63	22.73	0.996
	Sector 7	138.57	25.22	139.31	24.38	0.816
	Sector 8	123.40	22.11	127.00	20.26	0.183
	Sector 9	60.06	10.51	65.06	14.58	**0.005**
	Sector 10	51.77	10.30	52.82	11.25	0.462
	Sector 11	74.57	12.85	75.50	12.40	0.569
	Sector 12	116.59	22.98	119.38	24.38	0.372
**Macular thickness**	Fovea	270.99	23.98	266.57	22.13	0.135
	Inner superior	334.19	23.19	331.18	16.30	0.211
	Inner nasal	337.48	24.36	334.21	17.84	0.207
	Inner inferior	332.62	17.25	329.71	16.33	0.180
	Inner temporal	321.31	16.90	318.27	15.61	0.145
	Outer superior	287.84	15.91	284.96	13.85	0.127
	Outer nasal	306.75	18.44	304.61	17.16	0.348
	Outer inferior	277.40	16.15	276.19	13.43	0.518
	Outer temporal	272.09	14.86	270.37	12.12	0.312

In the second analysis, we divided the full population into two groups to evaluate the presence of atheroma plaques in carotid artery ultrasound scans. Group 1 comprised subjects with no evidence of atheroma on the ultrasound scan (97 subjects, 63.8% of the total subjects in the sample), and Group 2 comprised subjects with at least one plaque (55 subjects, 36.2% of the sample), regardless of the thickness and number of plaques found. Mean age and IOP for Group 1 were 50.40±3.54 years and 14.21±2.43 mmHg, respectively, and for Group 2, 51.28±3.72 years and 14.23±2.39 mmHg, respectively. The two groups were not significantly different with regard to age (p = 0.092) or IOP (p = 0.542). Mean OCT values for these two groups and significance level based on comparison with Student’s t test are shown in Table 2. The two groups did not differ significantly with regard to the RNFL measurements or macular structure analysis.

**Table 2:** Mean, standard deviation (SD), and comparison of retinal nerve fiber layer (RNFL) and macular thickness values obtained with the Cirrus High Definition optical coherence tomography (OCT) device between the group without atheroma plaques and the group with at least one atheroma plaque.

OCT measurements		NO ATHEROME PLAQUES IN ULTRASOUND (n = 97)		AT LEAST ONE ATHEROMA PLAQUE IN ULTRASOUND (n = 55)		SIGNIFICANCE (P)
		Mean	SD	Mean	SD	
**RNFL thickness**	Average	94.23	9.60	94.27	10.51	0.973
	Superior	116.78	14.86	118.20	20.47	0.513
	Nasal	72.81	12.21	72.97	14.27	0.918
	Inferior	123.48	16.15	122.41	14.91	0.559
	Temporal	64.04	10.93	63.70	10.06	0.782
	Sector 1	120.48	22.59	122.91	30.77	0.458
	Sector 2	105.68	18.39	109.13	28.91	0.247
	Sector 3	89.62	17.15	88.91	23.05	0.773
	Sector 4	59.28	11.42	59.93	11.72	0.633
	Sector 5	69.60	14.39	69.93	15.59	0.854
	Sector 6	104.21	21.79	103.24	23.63	0.721
	Sector 7	140.16	26.56	138.42	23.20	0.549
	Sector 8	126.83	22.84	125.49	19.37	0.584
	Sector 9	64.00	13.54	63.52	13.95	0.770
	Sector 10	52.28	10.70	52.71	11.21	0.739
	Sector 11	75.75	13.97	74.91	11.46	0.570
	Sector 12	121.23	25.45	116.91	22.91	0.126
**Macular thickness**	Fovea	268.46	21.80	267.31	23.33	0.670
	Inner superior	332.75	20.30	331.49	17.11	0.563
	Inner nasal	334.97	21.01	335.20	19.07	0.922
	Inner inferior	331.71	16.02	329.69	16.99	0.305
	Inner temporal	320.12	15.26	318.41	16.45	0.367
	Outer superior	287.05	14.18	284.86	14.64	0.201
	Outer nasal	306.11	17.36	304.57	17.63	0.459
	Outer inferior	277.48	14.13	275.88	14.27	0.342
	Outer temporal	272.18	13.14	269.91	12.72	0.139

The third analysis evaluated the homolateral effects on the eye depending on the laterality of the atheroma plaques. We compared retinal and RNFL structures in the right eyes of the subjects with and without signs of atheroma plaques in the right carotid. The same analysis was performed for the left carotid territory and left eye. In subjects with signs of atheroma plaques in the right carotid, we found a significant reduction in the RNFL superior quadrant (115.17±15.20 μm) compared with subjects without atheroma plaques in the right carotid (123.41±33.10 μm; p = 0.044), and also in the RNFL 2 o’clock sector (101.11±20.35 μm vs 114.56±43.81 μm; p = 0.014) (Table 3). In the left eye examination, a significant reduction was found in subjects with atheroma plaques in the left carotid compared with subjects without plaques in the central macular thickness (266.49±19.18 vs 276.89±23.66 μm; p = 0.016), in the RNFL superior quadrant (116.21±15.23 vs 124.89±15.23 μm; p = 0.006), and in RNFL 11 o’clock sector (73.18±12.13 vs 79.07± 10.72 μm; p = 0.018). The RNFL 11 o’clock sector is located in the superior pole of the optic disc (Table 4). We repeated the analysis using any atheroma plaques in any left-sided location (carotid and femoral arteries) for the left eye, and similarly for right-sided territories and the right eye. The results were similar to those found with respect to the carotid arteries. Lastly, the sub-analysis of subjects with atheroma plaques in the common carotid artery revealed similar results to those found in subjects with atheroma plaques in the left carotid. These group of individuals showed reduced measurements in the left eye compared to the right eye in the central macular thickness (p = 0.016), the superior RNFL quadrant (p = 0.007), and the 11 o’clock RNFL sector (p = 0.020).

**Table 3:** Mean, standard deviation (SD), and comparison of retinal nerve fiber layer (RNFL) and macular thickness values obtained with the Cirrus High Definition optical coherence tomography (OCT) device in the right eye, between the group with and without atheroma plaques in the right carotid.

OCT measurements		Subjects without atheroma plaques in the right carotid		Subjects with atheroma plaques in the right carotid		Significance (P)
		Mean	SD	Mean	SD	
**RNFL thickness**	Average	94.23	9.38	96.59	13.59	0.254
	Superior	115.17	15.19	123.41	33.09	***0*.*044***
	Nasal	73.83	12.67	74.74	19.311	0.750
	Inferior	122.67	15.15	125.26	15.21	0.384
	Temporal	65.59	12.20	63.44	8.04	0.337
	Sector 1	120.15	23.44	130.85	45.88	0.072
	Sector 2	101.11	20.35	114.56	43.81	***0*.*014***
	Sector 3	90.53	18.18	93.85	37.87	0.483
	Sector 4	60.22	11.70	61.50	14.55	0.600
	Sector 5	70.66	14.65	68.68	13.96	0.486
	Sector 6	102.19	21.13	105.82	23.26	0.392
	Sector 7	136.94	23.45	142.41	24.30	0.239
	Sector 8	128.74	22.79	127.65	17.93	0.798
	Sector 9	66.46	15.73	64.97	10.93	0.606
	Sector 10	53.32	13.23	52.82	10.35	0.841
	Sector 11	76.93	12.81	72.24	11.16	0.056
	Sector 12	119.34	23.91	119.71	25.32	0.939
**Macular thickness**	Fovea	268.52	24.32	263.94	25.39	0.348
	Inner superior	333.07	15.12	328.61	23.40	0.195
	Inner nasal	335.76	17.21	332.73	24.44	0.424
	Inner inferior	331.09	15.82	328.73	22.05	0.495
	Inner temporal	319.47	14.45	317.82	22.11	0.613
	Outer superior	285.66	14.28	283.70	15.70	0.499
	Outer nasal	305.55	15.96	304.12	20.77	0.674
	Outer inferior	276.34	14.56	277.70	15.03	0.641
	Outer temporal	271.20	12.13	270.88	14.82	0.900

**Table 4:** Mean, standard deviation (SD), and comparison of retinal nerve fiber layer (RNFL) and macular thickness values obtained with the Cirrus High Definition optical coherence tomography (OCT) device in the left eye, between the group with and without atheroma plaques in the left carotid.

OCT measurements		Subjects without atheroma plaques in the left carotid		Subjects with atheroma plaques in the left carotid		Significance (P)
		Mean	SD	Mean	SD	
**RNFL thickness**	Average	92.91	9.66	96.07	9.56	0.120
	Superior	116.21	15.23	124.89	15.29	***0*.*007***
	Nasal	71.73	12.18	71.46	13.14	0.920
	Inferior	121.66	15.66	124.46	16.21	0.398
	Temporal	62.05	9.17	63.82	8.75	0.355
	Sector 1	119.18	23.31	128.39	29.70	0.077
	Sector 2	110.42	21.40	116.11	21.99	0.210
	Sector 3	87.56	16.16	85.46	20.29	0.558
	Sector 4	58.43	10.75	58.89	10.58	0.838
	Sector 5	69.14	15.81	70.14	15.31	0.762
	Sector 6	103.43	22.71	106.93	28.41	0.487
	Sector 7	138.91	26.13	142.36	23.46	0.523
	Sector 8	123.44	19.47	123.96	20.86	0.900
	Sector 9	61.50	12.03	60.61	14.15	0.733
	Sector 10	51.52	9.75	52.07	6.99	0.777
	Sector 11	73.18	12.13	79.07	10.72	***0*.*020***
	Sector 12	115.58	23.34	121.36	22.24	0.236
**Macular thickness**	Fovea	266.49	19.18	276.89	23.65	***0*.*016***
	Inner superior	331.68	19.55	334.11	16.64	0.550
	Inner nasal	335.19	21.00	336.70	17.57	0.728
	Inner inferior	330.99	15.05	329.96	16.36	0.753
	Inner temporal	318.89	14.62	321.52	16.20	0.410
	Outer superior	285.33	13.48	288.78	17.51	0.261
	Outer nasal	304.92	16.96	305.11	20.77	0.959
	Outer inferior	276.66	13.41	275.30	15.64	0.645
	Outer temporal	270.26	12.31	271.70	16.35	0.607

In the fourth analysis, we divided the sample according to the cardiovascular risk calculated according to the Framingham criteria, creating Group 1 (comprising 114 subjects with no cardiovascular risk, 75% of the sample) and Group 2 (comprising 38 subjects with some degree of cardiovascular risk, 25% of the sample). Mean age and IOP were 52.61±3.80 years and 14.32±2.54 mmHg, respectively, for subjects that did not meet the Framingham criteria and 53.22±3.58 years and 14.12±2.38 mmHg, respectively, for subjects that did meet the Framingham cardiovascular criteria. Age and IOP were not significantly different between groups (p = 0.109 and 0.222, respectively). Mean OCT values for these two groups and the differences between groups based on Student’s t test are shown in Table 5. The two groups did not differ significantly with regard to the RNFL measurements or the macular structure analysis.

**Table 5:** Mean, standard deviation (SD), and comparison of retinal nerve fiber layer (RNFL) and macular thickness values obtained with the Cirrus High Definition optical coherence tomography (OCT) device in the group with and without cardiovascular risk according to the Framingham criteria.

OCT measurements		FRAMINGHAM NO CARDIOVASCULAR RISK (n = 114)		FRAMINGHAM CARDIOVASCULAR RISK (n = 38)		SIGNIFICANCE (P)
		Mean	SD	Mean	SD	
**RNFL thickness**	Average	93.92	10.01	94.31	10.23	0.776
	Superior	117.00	15.20	117.79	19.43	0.749
	Nasal	71.35	12.24	73.38	13.81	0.257
	Inferior	124.17	15.40	122.33	15.41	0.370
	Temporal	63.21	11.46	64.00	10.06	0.572
	Sector 1	121.47	22.72	122.06	29.29	0.873
	Sector 2	105.59	22.16	108.52	26.21	0.385
	Sector 3	87.31	17.70	89.85	21.79	0.361
	Sector 4	59.08	10.89	59.80	11.78	0.642
	Sector 5	67.52	13.88	70.47	15.41	0.142
	Sector 6	105.19	22.67	102.97	22.81	0.465
	Sector 7	139.60	23.44	138.92	25.06	0.835
	Sector 8	127.59	21.73	125.51	20.50	0.454
	Sector 9	63.49	14.59	63.75	13.50	0.888
	Sector 10	51.83	11.94	52.73	10.71	0.539
	Sector 11	74.47	12.68	75.46	12.50	0.553
	Sector 12	118.15	25.73	118.59	23.55	0.890
**Macular thickness**	Fovea	267.84	18.55	267.89	23.92	0.988
	Inner superior	332.25	14.71	331.86	19.56	0.872
	Inner nasal	335.60	16.54	334.95	20.87	0.807
	Inner inferior	331.08	15.29	330.31	17.06	0.729
	Inner temporal	320.52	14.38	318.53	16.52	0.352
	Outer superior	285.08	14.12	285.91	14.63	0.667
	Outer nasal	304.08	16.85	305.55	17.77	0.530
	Outer inferior	277.25	14.32	276.24	14.21	0.596
	Outer temporal	271.13	13.19	270.72	12.85	0.810

**Fig 1:**
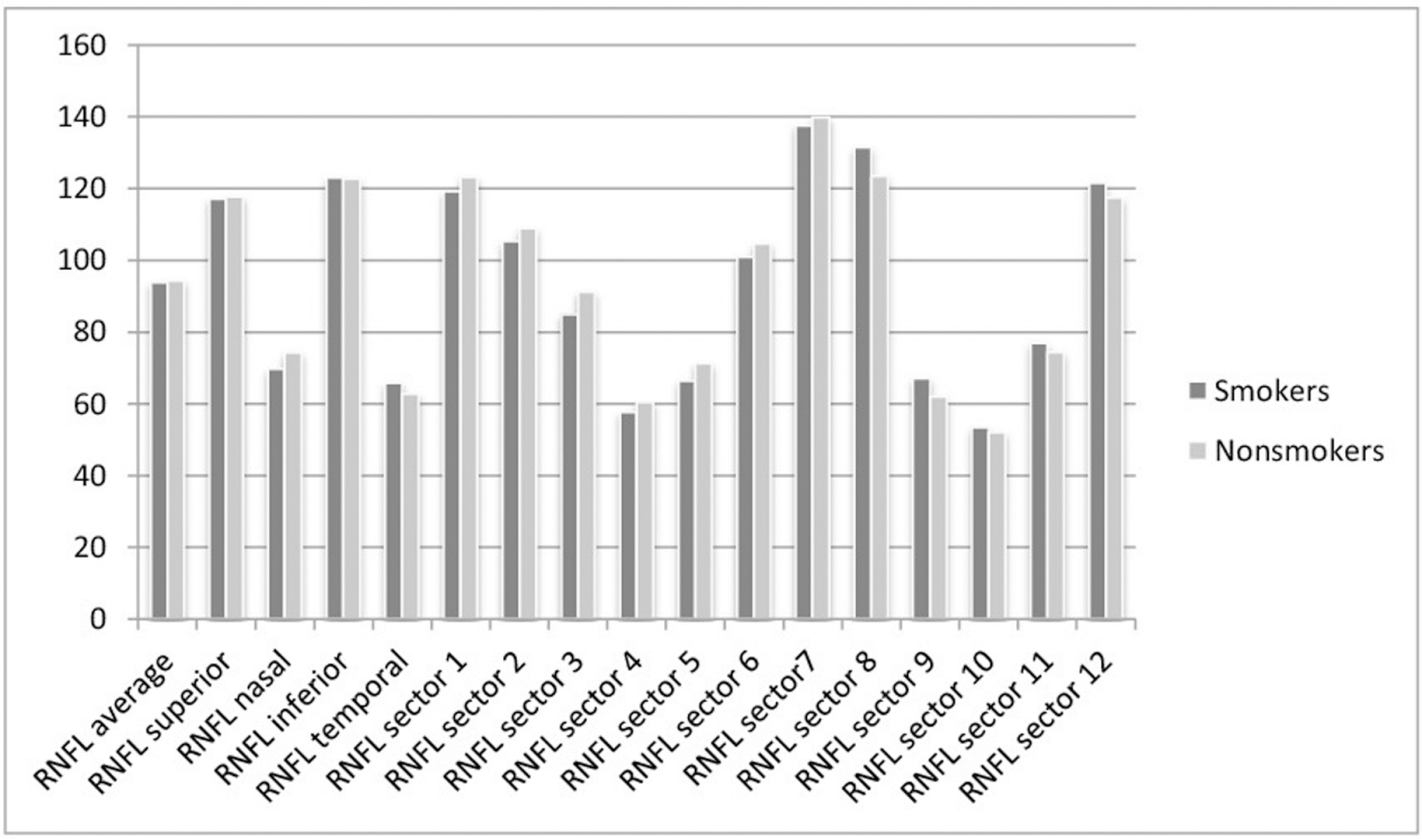
Bar graphs of optical coherence tomography measurements in microns. Representation in bar graphs of retinal nerve fiber layer (RNFL).

**Fig 2:**
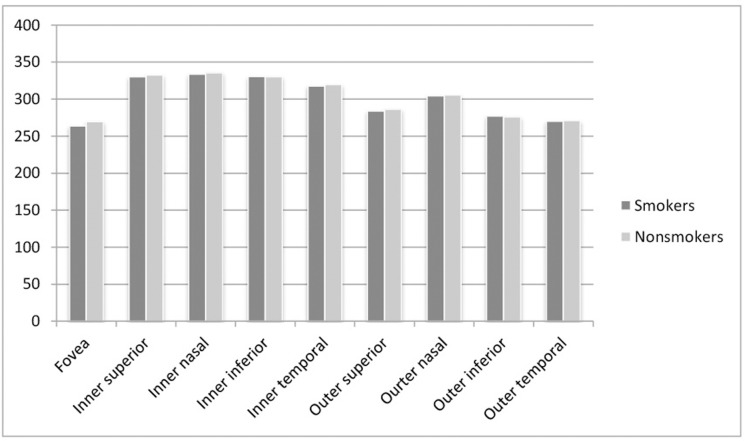
Bar graphs of optical coherence tomography measurements in microns. Representation in bar graphs of macular thickness values obtained with the Cirrus High Definition optical coherence tomography device in smokers and nonsmokers.

In the fifth analysis, we divided the sample into 92 nonsmokers and 47 smokers. We excluded 13 subjects whose smoking habit was unknown. Mean age of the nonsmokers was 51.35±3.81 years and that of the smokers was 51.11±3.48 (p = 0.596). Mean IOP values of the nonsmokers was 14.13±2.45 and for the smokers, 14.16±2.55 (p = 0.709). Figs 1 and 2 show the OCT measurement values for the smokers and nonsmokers groups. The central macular thickness was significantly thinner in the smokers (263.82±21.09 μm vs 269.78±23.16 μm; p = 0.034). A significant reduction was also detected in smokers in the RNFL nasal quadrant (69.77±12.16 vs 74.31±13.79 μm; p = 0.006), RNFL 3 o’clock sector (84.98±17.77 vs 91.17±21.88 μm; p = 0.016), and RNFL 5 o’clock sector (66.38±13.62 vs 71.28±15.48 μm; p = 0.009).

Finally, the multivariate regression analysis demonstrated that RNFL or macular parameters are not predictors of presence of classic cardiovascular risk factors or subclinical atherosclerosis in our population.

## Discussion

Because the RNFL comprises the first axons of the visual pathway and is unmyelinated, it can be considered a unique anatomic model that may provide insight into the pathophysiologic processes of neurodegenerative diseases. In fact, previous OCT studies have emphasized the role of the visual pathway as an ideal structure for exploring neurodegeneration and have demonstrated the potential of the method to longitudinally monitor structural changes in neurologic disorders such as multiple sclerosis, Alzheimer disease, Parkinson’s disease, and also some psychiatric diseases such as schizophrenia [12]. The spatial resolution of OCT scans is comparable to that of histologic slices [13].

Decreased RNFL thickness may reflect retinal neuronal-ganglion cell death. In the sectorial analysis of the RNFL, a deeper reduction in the temporal quadrant thickness compared to the other three quadrants is observed in neurodegenerative diseases [14–16]. Also, temporal RNFL thickness is reduced in patients with some mitochondrial disorders, including Leber's hereditary optic neuropathy and other neurologic conditions. This predominantly temporal reduction of the RNFL thickness seems to be consistent in the neuro-ophthalmologic literature, highlighting its usefulness as a biomarker of neurodegeneration. On the other hand, glaucoma leads to a greater decrease in the inferior RNFL quadrant thickness [17], and patients with obstructive sleep apnea syndrome present with RNFL thinning in the nasal quadrant and choroidal thickening (this change may be caused by the pathophysiology of the neurodegeneration process in obstructive sleep apnea syndrome) [18]. Therefore, each pathology may lead to a different fingerprint of damage in the optic nerve [19].

Cognitive impairment and neurologic degeneration are directly linked to chronic microvascular ischemic changes in the brain, which are often related to cardiovascular risk factors, such as cholesterol serum levels [20–22]. Many diseases are characterized by impaired cholesterol turnover in the brain. Cholesterol is important for synaptic transmission, and there is a link between cholesterol metabolism defects and neurodegenerative disorders [23,24].

In our study, we evaluated the implications and possible contributions of classic cardiovascular risk factors to subclinical alterations of the RNFL. The quantitative relationship between cardiovascular factors and risk of cardiovascular events was elucidated by the Framingham Heart Study and other studies [25]. These studies demonstrated that the major risk factors are additive in predictive power. Accordingly, a person’s total risk can be estimated by summing the risk afforded by each of the major risk factors. The American Heart Association and the American College of Cardiology published joint recommendations for medical interventions in patients with cardiovascular risk and other forms of atherosclerotic disease [26]. A similar potential exists for risk reduction in patients without an established cardiovascular risk (primary prevention). The risk status of persons without cardiovascular risk varies greatly.

The effects of cigarette smoking on RNFL measurements were analyzed, and a significant tendency toward nasal quadrant thinning of the RNFL was observed in smokers compared to nonsmokers. Thus, we postulate that tobacco affects the blood vessels in the deep retinal structures and that early thinning in the nasal area of the optic head nerve in the OCT scans might suggest other than neurodegenerative damage.

Our results revealed a significant reduction in the superior quadrant of the RNFL in the right eye of subjects with atheroma plaques in the right carotid artery territory. The same thinning was observed in the superior pole of the left optic disc in subjects with some degree of left carotid artery stenosis. Subjects with common carotid artery atheroma plaques presented a thinner superior area of the RNFL in the left eye. A potential explication of this finding may be that superior areas of the RNFL are more sensitive to be damaged by minimum blood flow defects, especially in the left eye. Ocular blood hypoperfusion is associated with many ophthalmic disorders, such as neovascular glaucoma, ocular ischemic syndrome, and vascular occlusions; and these disorders usually cause early defects in the superior area of the RNFL [27]. Defects in retinal ganglion cells are reported in patients with ictus and brain hypoxia, although optic atrophy and retinal nerve fiber layer defects may not be detected on fundoscopy, and these defects are more clearly detected by OCT than circumpapillary retinal nerve fiber layer defects. Transneuronal retrograde degeneration has been demonstrated [28,29]. As the ophthalmic artery is a branch of the internal carotid, and thus responsible for vascularization of the main structures in the eye, microvascular ischemia and carotid stenosis may lead to choroidal hypoperfusion, attenuation of the choroidal vessels, and alterations in the integrity of the RNFL, as measured by OCT [30].

To our knowledge, this is the first study to demonstrate a direct relation between subclinical stenosis of the carotid arteries and its branches measured by supra-aortic ultrasound scans, and the retinal structure analysis measured by OCT. These findings suggest that general screening with OCT in cases of suspected stenosis could be valuable to support the diagnosis or early detection of subclinical atrophy in the RNFL. This reduction in the RNFL seems to be selective for the superior quadrant–a remarkable fact given the RNFL alterations described in other neurologic conditions seems to be found preferentially in the temporal quadrant. Nevertheless, when OCT detects subclinical thinning in other areas of the retinal structure (outside the superior quadrant), this thinning should not be exclusively attributed to chronic ischemia and we should search for any neurodegenerative diseases or glaucoma, entities for which retinal abnormalities measured by OCT have been widely described.

We evaluated atheromatous plaques in the carotid, or intimal thickening, but no significant stenosis was detected in any subject. A possible explanation is that incipient and subclinical atherosclerosis in the carotid may extend distally to the arterioles and capillaries, including the eye area. Another possible explanation is that structural changes of atherosclerosis in this very early stage also affect other metabolic or molecular structures, such as neuroretinal areas.

A limitation of this study is that only men were included. Women were excluded from recruitment in an attempt to avoid selection bias because men are more frequently affected by cardiovascular conditions. Another possible limitation is the sample size because a larger sample would allow for different subgroups to be established for independent evaluation of each risk factor (e.g., smoke habit only, without diabetes, dyslipidemia, etc.). Further investigation is required to clarify the effect of sex and to evaluate a larger sample.

## References

[1] Garcia-Martin E, Pueyo V, Almarcegui C, Martin J, Ara JR, Sancho E, et al Risk factors for progressive axonal degeneration of the retinal nerve fibre layer in multiple sclerosis patients. Br J Ophthalmol 2011;95:1577–1582. doi: 10.1136/bjo.2010.199232 21785155 10.1136/bjo.2010.199232

[2] Garcia-Martin E, Pablo LE, Herrero R, Satue M, Polo V, Larrosa JM, et al Diagnostic ability of a linear discriminant function for Spectral domain optical coherence tomography in multiple sclerosis patients. Ophthalmology 2012;119:1705–1711. doi: 10.1016/j.ophtha.2012.01.046 22480742 10.1016/j.ophtha.2012.01.046

[3] Martinez-Lapiscina EH, Arnow S, Wilson J, Saidha S, Preiningerova JL, Oberwahrenbrock T, et al; IMSVISUAL consortium. Retinal thickness measured by optical coherence tomography and risk of disability worsening in multiple sclerosis. Lancet Neurol 2016;15:574–584. doi: 10.1016/S1474-4422(16)00068-5 27011339 10.1016/S1474-4422(16)00068-5

[4] Larrosa JM, Garcia-Martin E, Bambo MP, Pinilla J, Polo V, Otin S, et al Potential new diagnostic tool for Alzheimer’s disease using a linear discriminant function for Fourier domain optical coherence tomography. Invest Ophthalmol Vis Sci 2014;55:3043–3051. doi: 10.1167/iovs.13-13629 24736054 10.1167/iovs.13-13629

[5] Garcia-Martin E, Satue M, Otin S, Fuertes I, Alarcia R, Larrosa JM, et al Retina measurements for diagnosis of Parkinson disease. Retina 2014;34:971–980. doi: 10.1097/IAE.0000000000000028 24172914 10.1097/IAE.0000000000000028

[6] Casasnovas JA, Alcaide V, Civeira F, Guallar E, Ibañez B, Borreguero JJ, et al Aragon workers' health study—design and cohort description. BMC Cardiovasc Disord 2012;12:45 doi: 10.1186/1471-2261-12-45 22712826 10.1186/1471-2261-12-45 PMC3439398

[7] Kure CE, Chan YK, Ski CF, Thompson DR, Carrington MJ, Stewart S. Gender-specific secondary prevention? Differential psychosocial risk factors for major cardiovascular events. Open Heart 2016;3:e000356 doi: 10.1136/openhrt-2015-000356 27099759 10.1136/openhrt-2015-000356 PMC4836286

[8] Laclaustra M, Casasnovas JA, Fernández-Ortiz A, Fuster V, León-Latre M, Jiménez-Borreguero LJ, et al Femoral and carotid subclinical atherosclerosis association with risk factors and coronary calcium: the AWHS study. J Am Coll Cardiol 2016;67:1263–1274. doi: 10.1016/j.jacc.2015.12.056 26988945 10.1016/j.jacc.2015.12.056

[9] Stein JH, Korcarz CE, Hurst RT, Lonn E, Kendall CB, Mohler ER, et al Use of carotid ultrasound to identify subclinical vascular disease and evaluate cardiovascular disease risk: a consensus statement from the American Society of Echocardiography Carotid Intima-Media Thickness Task Force. J Am Soc Echocardiogr 2008;21:93–111. doi: 10.1016/j.echo.2007.11.011 18261694 10.1016/j.echo.2007.11.011

[10] Agatston AS, Janowitz WR, Hildner FJ, Sheedy PF, Breen JF, Rumberger JA. Quantification of coronary artery calcium using ultrafast computed tomography. J Am Coll Cardiol 1990;15:827–832. 2407762 10.1016/0735-1097(90)90282-t

[11] Schuman JS, Pedut-Kloizman T, Hertzmark E, Hee MR, Wilkins JR, Coker JG, et al Reproducibility of nerve fiber layer thickness measurements using optical coherence tomography. Ophthalmology 1996;103:1889–1898. 8942887 10.1016/s0161-6420(96)30410-7 PMC1939724

[12] Jones-Odeh E, Hammond CJ. How strong is the relationship between glaucoma, the retinal nerve fibre layer, and neurodegenerative diseases such as Alzheimer's disease and multiple sclerosis? Eye (Lond) 2015;29:1270–1284. 26337943 10.1038/eye.2015.158 PMC4815693

[13] Garcia-Martin E, Pablo LE, Gazulla J, Vela A, Larrosa JM, Polo V, et al Retinal segmentation as noninvasive technique to demonstrate hyperplasia in ataxia of Charlevoix-Saguenay. Invest Ophthalmol Vis Sci 2013;54(10):7137–7142. doi: 10.1167/iovs.13-12726 24114536 10.1167/iovs.13-12726

[14] Salobrar-Garcia E, Hoyas I, Leal M, de Hoz R, Rojas B, Ramirez AI, et al Analysis of retinal peripapillary segmentation in early Alzheimer's disease patients. Biomed Res Int 2015;2015:636548 doi: 10.1155/2015/636548 26557684 10.1155/2015/636548 PMC4628738

[15] Kersten HM, Danesh-Meyer HV, Kilfoyle DH, Roxburgh RH. Optical coherence tomography findings in Huntington's disease: a potential biomarker of disease progression. J Neurol 2015;262:2457–2465. doi: 10.1007/s00415-015-7869-2 26233693 10.1007/s00415-015-7869-2

[16] Ewering C, Haşal N, Alten F, Clemens CR, Eter N, Oberwahrenbrock T, et al Temporal retinal nerve fibre layer thinning in cluster headache patients detected by optical coherence tomography. Cephalalgia 2015;35:946–958. doi: 10.1177/0333102414560632 25657327 10.1177/0333102414560632

[17] Hwang YH, Kim YY. Application of the ISNT rule to neuroretinal rim thickness determined using Cirrus HD optical coherence tomography. J Glaucoma 2015;24:503–507. doi: 10.1097/IJG.0000000000000015 24240880 10.1097/IJG.0000000000000015

[18] Ozge G, Dogan D, Koylu MT, Ayyildiz O, Akincioglu D, Mumcuoglu T, et al Retina nerve fiber layer and choroidal thickness changes in obstructive sleep apnea syndrome. Postgrad Med 2016;128:317–322. doi: 10.1080/00325481.2016.1159118 26918297 10.1080/00325481.2016.1159118

[19] Satue S, Obis J, Rodrigo MJ, Otin S, Fuertes MI, Vilades E, et al Retinal measurements as biomarkers for diagnosis, progression and prognosis of neurodegenerative diseases. J Ophthalmol 2016 In press. 10.1155/2016/8503859 PMC5093273 27840739

[20] Kalesnykas G, Tuulos T, Uusitalo H, Jolkkonen J. Neurodegeneration and cellular stress in the retina and optic nerve in rat cerebral ischemia and hypoperfusion models. Neuroscience 2008;155:937–947. doi: 10.1016/j.neuroscience.2008.06.038 18640247 10.1016/j.neuroscience.2008.06.038

[21] Akay F, Gündoğan FC, Yolcu U, Toyran S, Uzun S. Retinal structural changes in systemic arterial hypertension: an OCT study. Eur J Ophthalmol 2016;26(2):152–7. doi: 10.5301/ejo.5000675 26951532 10.5301/ejo.5000740

[22] Shariati MA, Park JH, Liao YJ. Optical coherence tomography study of retinal changes in normal aging and after ischemia. Invest Ophthalmol Vis Sci 2015;56:2790–2797. doi: 10.1167/iovs.14-15145 25414186 10.1167/iovs.14-15145

[23] Petrov AM, Kasimov MR, Zefirov AL. Brain cholesterol metabolism and its defects: linkage to neurodegenerative diseases and synaptic dysfunction. Acta Naturae 2016;8:58–73. 27099785 PMC4837572

[24] Liu QY, Koukiekolo R, Zhang DL, Smith B, Ly D, Lei JX, et al Molecular events linking cholesterol to Alzheimer's disease and inclusion body myositis in a rabbit model. Am J Neurodegener Dis 2016;5:74–84. 27073745 PMC4788734

[25] Wilson PW, D’Agostino RB, Levy D, Belanger AM, Silbershatz H, Kannel WB. Prediction of coronary heart disease using risk factor categories. Circulation 1998;97:1837–1847. 9603539 10.1161/01.cir.97.18.1837

[26] Smith SC Jr, Blair SN, Criqui MH, Fletcher GF, Fuster V, Gersh BJ, et al; Secondary Prevention Panel. Preventing heart attack and death in patients with coronary disease. Circulation 1995;92:2–4. 7788911

[27] Lyons Wait VA, Anderson SF, Townsend JC, De Land P. Ocular and systemic findings and their correlation with hemodynamically significant carotid artery stenosis: a retrospective study. Optom Vis Sci 2002;79:353–362. 12086301 10.1097/00006324-200206000-00008

[28] Park HY, Park YG, Cho AH, Park CK. Transneuronal retrograde degeneration of the retinal ganglion cells in patients with cerebral infarction. Ophthalmology 2013;120:1292–1299. doi: 10.1016/j.ophtha.2012.11.021 23395544 10.1016/j.ophtha.2012.11.021

[29] Kim M, Park KH, Kwon JW, Jeoung JW, Kim TW, Kim DM. Retinal nerve fiber layer defect and cerebral small vessel disease. Invest Ophthalmol Vis Sci 2011;52:6882–6886. doi: 10.1167/iovs.11-7276 21791593 10.1167/iovs.11-7276

[30] Yamashita T, Miki A, Iguchi Y, Kimura K, Maeda F, Kiryu J. Reduced retinal ganglion cell complex thickness in patients with posterior cerebral artery infarction detected using spectral-domain optical coherence tomography. Jpn J Ophthalmol. 2012;56:502–510. doi: 10.1007/s10384-012-0146-3 22684619 10.1007/s10384-012-0146-3

